# Biosimilars in the management of chronic inflammatory diseases: The Dutch experience

**DOI:** 10.31138/mjr.30.1.76

**Published:** 2019-05-31

**Authors:** Wieland D. Müskens, Sanne A. A. Rongen-van Dartel, Eddy M. M. Adang, Piet L. C. M. van Riel

**Affiliations:** 1 Department of IQ Healthcare, Radboudumc, Nijmegen, The Netherlands,; 2 Department of Rheumatology, Bernhoven, Uden, The Netherlands,; 3 Department of Health Evidence, Radboudumc, Nijmegen, The Netherlands

**Keywords:** Biosimilars, nocebo-effect, switching, biologicals, non-medical switch

## Abstract

These days, the use of biosimilars for the treatment of bio-naive patients is well established. However, the transition of patients being treated with a bio-originator to its biosimilar is still a topic of discussion. The main issue is which approach to use when initiating the non-medical transition. The first real-world examples contain both mandatory and non-mandatory approaches, resulting in a variety of acceptance and discontinuation rates. At this moment a non-mandatory approach, based on shared decision making, is preferred by international guidelines and the Task Force on the Use of Biosimilars to Treat Rheumatological Diseases. However, clear definitions of mandatory and non-mandatory are lacking, as a result of which these terms may be wrongly used in some studies. This article aims to provide an overview of transition approaches used in the Netherlands, and how the approach used relates to acceptance and discontinuation rates of the biosimilar.

## INTRODUCTION

In 2015, the Dutch Medicines Evaluation Board updated their point of view on biosimilars.^1^ From that moment on, patients being treated with a bio-originator could be transitioned to its biosimilar, if the patients were adequately monitored and well informed. Before that time, the Dutch Medicines Evaluation Board was of the opinion that patients should be kept on a biological medicinal product as much as possible, and strongly advised that biosimilars should only be prescribed to bio-naïve patients. As an addendum, the Federation of Medical Specialists (FMS) stated that patient should undergo only one switch between bio-originator and biosimilar and that repeated exchange between biological drugs had to be avoided.^2^

One of the fields where biologicals are widely prescribed is in the management of inflammatory rheumatic diseases. In 2015, more than 300 million euro were spent on this in the Netherlands alone, with an average of more than 10.000 euro per patient per year.^3^

In the Netherlands, prices for biologicals are negotiated at the hospital or at the regional level. Therefore, it varies among hospitals and regions, whether the bio-originator or the biosimilar gives the best value for money. A good example of this is the bio-originator for adalimumab, which after the expiration of its patent, is offered at an 80% discount.^4^ These kinds of discounts influence both which biological bio-naïve patients receive and whether transitioning patients for non-medical reasons is feasible. Therefore, driven by their financial incentives, hospitals customize transitioning to fit their own situation. This results in hospitals that differ in whether they transition to biosimilars at all, which patients they offer transitioning to, and whether the transitioning is some sort of mandatory or not.

## BIOSIMILARS IN THE REAL WORLD

Though international studies show that the acceptance rate of transitioning to biosimilars is in general quite high, 79–99%,^5–7^ the retention rates after transitioning seem to differ between a blinded setting and data from daily clinical practice. A systematic review from 2018, investigating discontinuation after transitioning to biosimilars in inflammatory diseases, found a median discontinuation rate of 14% in open-label studies versus 7% in double-blinded settings.^8^ This difference is hypothesized to be due to the nocebo effect (the negative counterpart of the placebo phenomenon)^9^ and attribution effects (the undeserved allocation of pre-existing or unrelated symptoms to the new medication),^10^ though conclusive evidence for this is still lacking.^8,11^ At this point, most people believe that the nocebo effect can be reduced through better shared decision making and patient information.^12–14^ Therefore, emphasis is put on the process of transitioning, which offers an opportunity to inform patients, and “get them on board” with the process.

Because in the Netherlands each hospital customizes its transition, the Dutch situation gives us the opportunity to present an overview of different transitioning strategies and how these might influence outcome measures, like acceptance of transitioning, and the retention rate of treatment after transitioning.

## TRANSITIONING IN THE NETHERLANDS

A survey conducted in all Dutch hospitals in 2016 found that 87% of the hospitals have some sort of policy in place regarding the use of biosimilars. Of these hospitals, about 50% indicated that they transition patients on bio-originator treatment to a biosimilar.^15^ However, only a small number of hospitals has published data on transitioning to a biosimilar for the treatment of inflammatory diseases (*Table 1*).

**Table 1:** Transitioning to biosimilars for the treatment of inflammatory diseases in the Netherlands.

**Study (year) Ref. No.**	Approach^*^	**Biological**	**Patients**	**Diseases included**	**Acceptance rate (n)**	**Discontinuationrate (N)**	**Duration (months)**
Binkhorst et al. (2018)	Non-mandatory	Infliximab	256	CD, CU	77%(197)	10%(20)	4
Boone et al. (2018)	Non-mandatory	Infliximab	146	RA, PsA, AS CD, CU	86%(125)	18%(22)	9
Layegh et al. (2019)	Mandatory	Infliximab	47	RA, PsA	96% (45)	13%(6)	24
Müskens et al. (2018)	Non- mandatory	Etanercept	79	RA, PsA, AS	87% (69)	28%(19)	12
Schmitz et al. (2018)	Mandatory	Infliximab	133	CD, CU	100%(133)	26%(35)	12
Smits et al. (2019)	Mandatory	Infliximab	84	CD, CU	99%(83	6%(5)	4
-	-	-	-	-	-	18%(15)	12
-	-	-	-	-	-	34%(28)	24
Tweehuysen et al. (2018)	Non- mandatory	Infliximab	222	RA, PsA, AS	86%(192)	24%(47)	6
Tweehuysen et al. (2018)	Mandatory	Etanercept	642	RA, PsA, AS	99%(635)	10%(60)	6

CD= Crohn’s disease; CU= colitis ulcerosa; RA= Rheumatoid arthritis; PsA= psoriatic arthritis; AS= ankylosing spondylitis

* Whether an approach was mandatory was based on the description of the approach given in the paper and based on the two following definitions: 1. Mandatory transition: patients were informed that a transition to biosimilar was happening. Following that, they were automatically transitioned. Only if they actively objected to the transition, were they not transitioned. 2. Non-mandatory transition: patients were informed of the possibility to transition. They were not transitioned, except when they actively agreed to the transition.

### Method of transitioning and the acceptance rate

In total, 8 studies were identified (*Table 1*). However, even when data were published, it is not always stated whether the transition was mandatory or not, and when it is stated, there is no clear definition in what is called mandatory and non-mandatory. Therefore, we used the following two definitions and categorised the used approach by the description of transitioning given in the paper.

- Mandatory transition: patients were informed that a transition to biosimilar was happening. Following that, they were automatically transitioned. Only if they *actively objected* to the transition, they were not placed on the biosimilar.
- Non-mandatory transition: patients were informed of the possibility to transition. They were not transitioned, except when they *actively agreed*.

#### Mandatory transition

We identified four studies that used a mandatory approach, with an acceptance rate ranging from 96% to 100%.^7,16–20^ The studies by Smits et al.^16–18^ and Schmitz et al.^19^ did not state whether their transition was mandatory or not, but after examining their approach, both seem to have been mandatory. The transitioning by Smits et al. took place after careful patient counselling while maintaining medical directives as per hospital protocol. Next to that, they stated that all patients were switched regardless of disease activity. Schmitz et al. communicated by letter to all patients that the transition was happening. The few people who had severe doubts regarding transitioning were persuaded by the physician to transition after a thorough explanation of the biosimilar concept. After this procedure, all patients transitioned. Two other studies which used a mandatory approach, were performed by Tweehuysen et al.^7^ and by Layegh et al.^20^ They used a communication strategy developed by Tweehuysen et al.,^21^ which was later incorporated by the Dutch Association of Hospital Pharmacists (NVZA) into the NVZA “toolbox Biosimilars”, which is a practical guideline for transitioning to biosimilars.^21^ Key elements of the strategy are: uniform communication; strict protocols; positive framing, tailored information and a wait and see approach if subjective health complaints arise. Tweehuysen et al. named their approach non-mandatory.^7^ However, when examining the structured strategy more closely it becomes apparent that it better fits a mandatory approach. Their information letter stated that the hospital was transitioning to a biosimilar and that patients had the opportunity to ask the pharmaceutical assistant questions regarding delivery of the biosimilar, during the next delivery of the medication. If a patient refused the transition, the reasons why the hospital was transitioning were stressed again. If a patient still refused, their rheumatologist would make contact within 3 days to discuss the transitioning. If after this consultation the patients still did not want to transition, the patient could continue with the bio-originator therapy.^21^ It is very likely that the hurdles required to decline transition in this approach explains the near-complete acceptance.

#### Non-mandatory approach

Four other studies followed a non-mandatory approach and found acceptance rates varying from 77% to 87%.^22–25^ Of these four studies, two clearly stated they tried to incorporate shared decision making in their transitioning method. In the first study, by Boone et al.,^22^ after informing patients by written documentation of the expectations following transitioning, patients were offered oral clarification by the patients’ treating physician or nurse practitioner when requested. Next to that, they formalised the procedure of giving informed consent by the patient, to emphasize that the change of treatment was introduced in a shared decision-making context.^22^ In the second study, performed by Müskens et al.,^23^ all patients on bio-originators were informed by letter of the possibility to transition to biosimilars in the near future. During the next outpatient visit with their rheumatologist, the possibility to transition to a biosimilar was discussed with the patients treated with an etanercept bio-originator. If the patients’ disease was not stable according to their rheumatologist, they were not eligible for transitioning. In this case, change to another biological treatment was discussed. Patients had the opportunity to ask questions regarding biosimilars and the switch to a biosimilar. If patients agreed, the transition was made, with the reservation that they could switch back to the originator at any time if they encountered difficulties with the biosimilar.

The only study that does not state that patients were informed by letter about the transition was the study by Binkhorst et al.:^25^ they only stated that patients were asked to transition to the biosimilar, implying the discussion took place during a consultation. The transition was initiated after patients had given informed consent.

## DISCONTINUATION OF BIOSIMILAR THERAPY AFTER TRANSITIONING

Most studies did not specify how they approached (subjective) health complaints after transitioning. Also, the duration of follow-up did differ across studies. Therefore, it is difficult to assess differences in discontinuation rates and attribute this to the method of transitioning (*Table 1*). However, when looking at the discontinuation rates, the drop in discontinuation between the two transitions conducted by Tweehuysen et al.^7,24^ is noteworthy. They found a lower discontinuation after the transition of etanercept, compared to the transition of infliximab (10% versus 24% respectively after 6 months). A reason for the lower discontinuation rate was that they started using the aforementioned mandatory communication strategy in an attempt to prevent possible nocebo and attribution effects. Another aspect of the communication strategy was the “wait-and-see approach”. This meant that when patients experienced subjective health complaints, the physician discussed that this might be due to the nocebo effect. When the patients agreed, treatment with the biosimilar was continued. The study by Layegh et al.^20^ which used the same, mandatory, approach, observed similar low discontinuation rates (13% after 24 months). This approach differs strongly with the approach used by Müskens et al.,^23^ where it was clearly communicated before acceptance of the transitioning that patients were free to return to the originator if side effects occurred, or lack efficacy was perceived by the patients. This might be reflected in the higher discontinuation rate found by Müskens et al.(28% after 12 months).^23^

## DISCUSSION

A brief overview of the published results from the Netherlands shows that acceptance of transitioning is generally high, with acceptance rates higher than 77%. Most hospitals used a well-structured plan, with clear written patient information in combination with the possibility to consult with the treating physician or nurse practitioner. In several publications, the terms mandatory and non-mandatory transitioning were used. However, because these terms lack clear definitions, we could not use the term used by the authors. Based on the aforementioned definitions of a non-mandatory and mandatory transition, we re-categorised the different studies.

The main difference in acceptance rates for transitioning to biosimilars was explained by whether a hospital used a mandatory or non-mandatory approach. Mandatory methods resulted in the highest acceptance (96–100%), but this came at the expense of the shared decision-making process. There was a large difference in discontinuation rates between studies, which was difficult to compare because of differences in follow-up time. However, there seems to be a trend for higher retention rates when a mandatory approach is used, especially in case it is combined with a “wait-and-see” approach.

The structured communication strategy from Tweehuysen et al.^21^ as described above, resulted in near complete acceptance and relatively low discontinuation. Therefore, the NVZA included it in the “toolbox biosimilars”. However, this approach is clearly mandatory and does not promote shared decision-making between the patient and the physician. Personal communication from a small set of patients taking part in the transitioning from Etanercept originator to its biosimilar by Tweehuysen et al.^7^ mentioned that they did not feel like they had a choice about whether or not to switch.

This accurately describes the tension between financial incentives and promoting shared decision-making in case of transitioning to biosimilars for non-medical reasons. In fact, there seems to be a trade-off between both forces that manifests itself in a choice between a mandatory and non-mandatory approach. Patient awareness about biosimilars is generally low^26^ and individual patients do not benefit from transitioning to a biosimilar. At the same time, the use of biosimilars is expected to bring great economic benefits on the societal level.^27,28^ The Task Force on the Use of Biosimilars to Treat Rheumatological Conditions captured this tension in two over-arching principles:^14^

1. “Treatment of rheumatic diseases is based on a shared decision-making process between patients and their rheumatologists”
2. “The contextual aspects of the healthcare system should be taken into consideration when treatment decisions are made”.

They concluded that, “given the complex nature of all biopharmaceuticals, the treating clinician must be the only one to decide whether to prescribe a biosimilar in place of a bio-originator on a case-by-case basis with full awareness of the patient”.^14^

Current practice in the Netherlands, where most patients are transitioned using a mandatory method does not seem to adhere to these recommendations. Although maximal acceptance rates can be reached with the “toolbox biosimilars”, the question should be asked how this tool influences the shared decision-making process. As this tool does not promote shared decision making, it is interesting to evaluate the extent of shared decision making experienced by the patients.

At the same time, it should be evaluated what the effects of different transition approaches are on cost-reduction and healthcare resource utilisation. The fact that lower medication prices will lead to lower medication cost on an individual base seems straightforward. But scarce real-world data on post-transition reports increased in-patient readmission rates, increased steroid use, extra consultations, and increased biosimilar dosing.^29^ This should warn us that a non-medical transitioning could finally lead to an increase in the total costs.

Though transitioning to biosimilars is relatively new, and real-world data are scarce, lessons learned from mandatory transitioning to generics bring warning. In the Netherlands, chronic patients are transitioned to generics for non-medical reasons, with 37% transitioning as often as three times or more on a yearly basis. 78% of the patients find transitioning for non-medical reasons problematic, 34% felt worse after substitution and 23% had to undergo extra examination after substitution. At the same time, 40% of the respondents reported to suffer from side effects of the new medication.^30^ By taking shared decision-making out of the transition process in the field of biologicals, a similar experience might be observed here as well.

At the moment, biosimilars are being prescribed for the treatment of inflammatory rheumatic diseases in both bionaïve patients and in patients undergoing treatment with the bio-originator. There is a lot of heterogeneity in the approaches used for initiating the transition to biosimilars, but also in how subjective health complaints are handled when they arise. Lack of a clear definition of what non-mandatory transitioning is makes it impossible to blindly copy the wording used by authors. However, in the Netherlands, at present, there seems to be a preference for a mandatory approach resulting in higher acceptance rate and lower discontinuation rates. This comes at the expense of the shared decision-making process. It seems contradictory that policy makers, who are often advocating SDM, implement a policy that is primarily aimed at maximizing cost savings.
